# 5-Methyl-1,3-phenyl­ene bis­[5-(di­methyl­amino)­naphthalene-1-sulfonate]: crystal structure and DFT calculations

**DOI:** 10.1107/S2056989019009058

**Published:** 2019-06-28

**Authors:** Tanwawan Duangthongyou, Ramida Rattanakam, Kittipong Chainok, Songwut Suramitr, Thawatchai Tuntulani, Boontana Wannalerse

**Affiliations:** a Department of Chemistry and Center of Excellent for Innovation in Chemistry, Faculty of Science, Kasetsart University, Bangkok 10903, Thailand; b Department of Chemistry, Faculty of Science, Kasetsart University, Bangkok 10903, Thailand; c Materials and Textile Technology, Faculty of Science, and Technology, Thammasart University, Pathum Thani 12120, Thailand; d Center of Nanotechnology, Department of Chemistry, Faculty of Science, Kasetsart University, Bangkok 10903, Thailand; e Supramolecular Chemistry Research Unit, Department of Chemistry, Faculty of Science, Chulalongkorn University, Bangkok 10330, Thailand

**Keywords:** crystal structure, dansyl unit, 3,5-di­hydroxy­toluene, hydrogen bonds, DFT calculations

## Abstract

The title compound, C_31_H_30_N_2_S_2_O_6_, possesses crystallographically imposed twofold symmetry with the two C atoms of the central benzene ring and the C atom of its methyl substituent lying on the twofold rotation axis. The two dansyl groups are twisted away from the plane of methyl­phenyl bridging unit in opposite directions. The three-dimensional arrangement in the crystal is mainly stabilized by weak hydrogen bonds between the sulfonyl oxygen atoms and the hydrogen atoms from the *N*-methyl groups. Stacking of the dansyl group is not observed. From the DFT calculations, the HOMO–LUMO energy gap was found to be 2.99 eV and indicates *n*→π* and π→π* transitions within the mol­ecule.

## Abstract 2

The title compound possesses crystallographically imposed twofold symmetry with the two C atoms of the central benzene ring and the C atom of its methyl substituent lying on the twofold rotation axis. The two dansyl groups are twisted away from the plane of methyl­phenyl bridging unit in opposite directions. The crystal packing features weak C—H⋯O hydrogen bonds.

## Chemical context   

Dansyl probes play important roles in many fields, including their use as industrial tracers and labelled biological tags (Tondi *et al.*, 2005 ▸; Li *et al.*, 2006 ▸; Liu *et al.*, 2016 ▸). Dansyl derivatives have been employed to identify some diseases within cells and to detect DNA-duplex sequences. For example, modified oligonucleotides that contain a dansyl fluoro­phore and (*S*)-2, 3-dihy­droxy propyl carbamates linked to guanine residues result in an enhancement of the fluorescence. Such modified oligonucleotides can be used to prepare and detect the sequence of fluoro­genic probes in DNA (Suzuki *et al.*, 2013 ▸). Cu-labelled dansyl mol­ecules have been designed and synthesized as fluorescence probes for membrane tags on apoptosis cells. These compounds can also be used for PET imaging of the apoptosis *in vivo* (Han *et al.*, 2016 ▸). Furthermore, the development of dansyl fluoro­genic receptors for cations, anions and neutral mol­ecules has attracted much attention because of their ability to turn fluorescence ‘on’ or ‘off’ through a number of mechanisms including ICT, PET and ET processes (Chen & Chen, 2005 ▸; Praveen *et al.*, 2010 ▸; Dinake *et al.* 2012 ▸; Jeong *et al.* 2016 ▸). In this paper, we report the synthesis, mol­ecular structure and crystal packing of 5-methyl-1,3-phenyl­ene bis­[5-(di­methyl­amino)­naphthalene-1-sulfonate]. The results of DFT calculations on the mol­ecule are also reported.
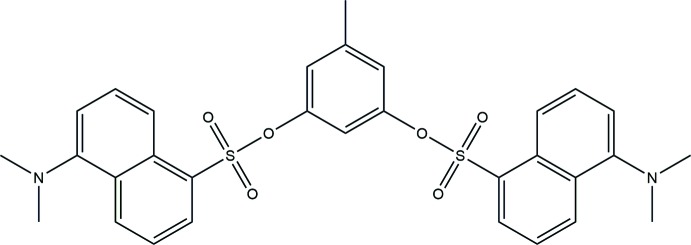


## Structural commentary   

**Figure 1:**
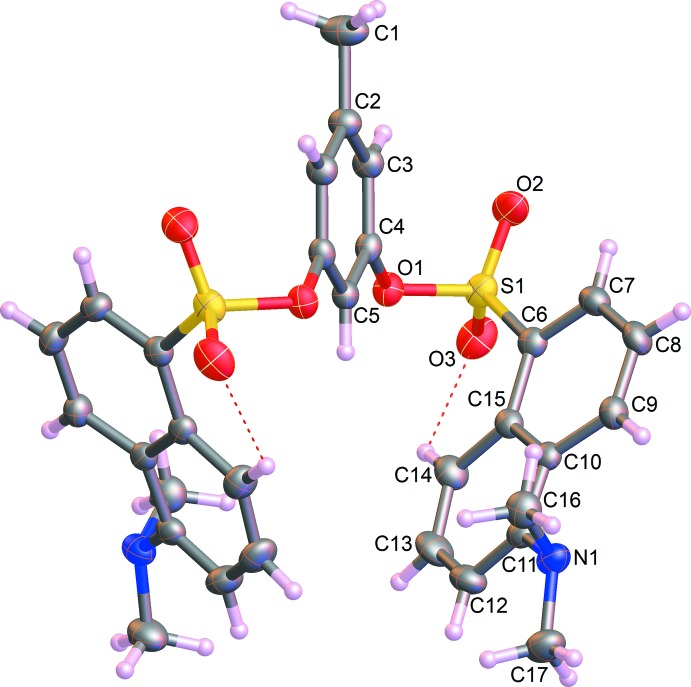
The mol­ecular structure of the title compound with displacement ellipsoids are drawn at the 50% probability level. Intra­molecular hydrogen bonds are shown as red dashed lines.

The title compound crystallizes in the space group *C*2/*c*. The mol­ecule lies on a crystallographic twofold axis running through atoms C1, C2 and C5 of the methyl­phenyl unit so that the asymmetric unit comprises one half-mol­ecule (Fig. 1 ▸). The hydrogen atoms of the C1 methyl group are therefore disordered over two equivalent positions. Intra­molecular C14—H14—O3 hydrogen bonds enclose *S*(6) rings, Fig. 1 ▸. The mol­ecular structure comprises two *O*-dansyl groups on either side of a bridging methyl­phenyl ring that is essentially planar. The S1—O1—C4—C3 torsion angle is 72.98 (16)° with the methyl­phenyl ring plane. The S1 sulfur atoms have distorted tetra­hedral geometries, with an O2—S1—C6 bond angle of 109.18 (8)°. The two naphthalene units in each dansyl group are inclined to one another at an angle of 52.29 (6)°; however, no stacking of the naphthalene units is observed.

## Supra­molecular features   

**Figure 2:**
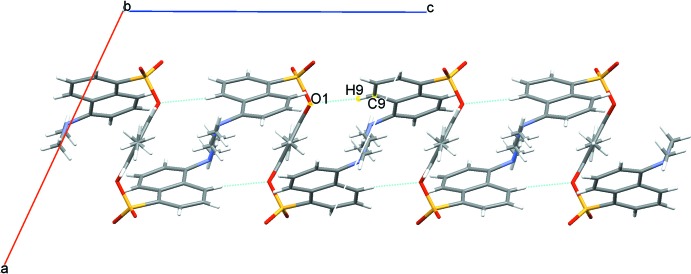
Chains of dimers of the title compound along the *c* axis. Dashed lines represent the C—H⋯O hydrogen bonds.

**Figure 3:**
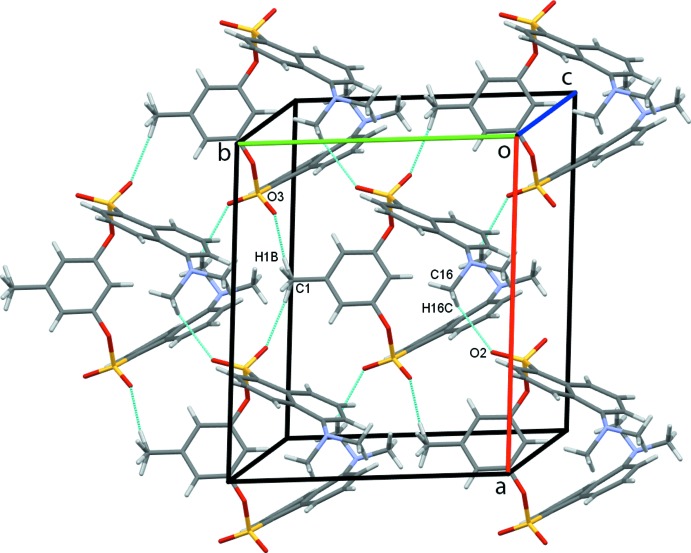
Sheets of mol­ecules of the title compound formed in the *ab* plane.

**Figure 4:**
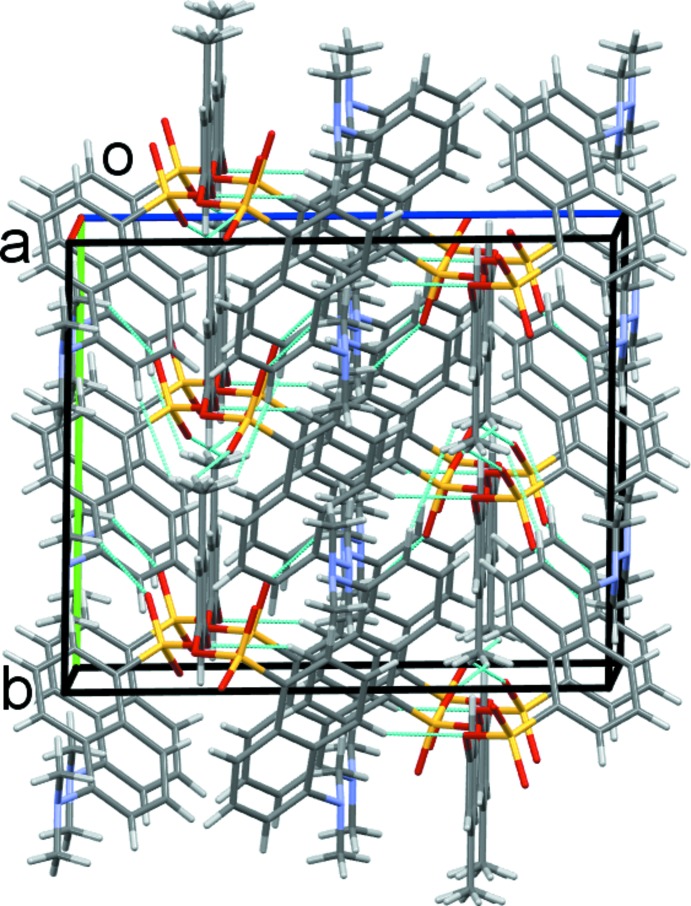
The overall packing of the title compound viewed along the *a*-axis direction.

In the crystal structure, the supra­molecular packing is dominated by weak C—H⋯O hydrogen bonds, Table 1 ▸. C9—H9—O1 contacts form dimers enclosing 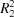
(22) rings and generate chains of mol­ecules along the *c*-axis direction, Fig. 2 ▸. C1—H1*B*—O3 and C16—H16*C*—O2 contacts further link the mol­ecules into sheets in the *ab* plane, Fig. 3 ▸. These contacts combine to stack rows of mol­ecules arranged in an obverse fashion along the *a*-axis direction, Fig. 4 ▸.

## Computational study   

**Figure 5:**
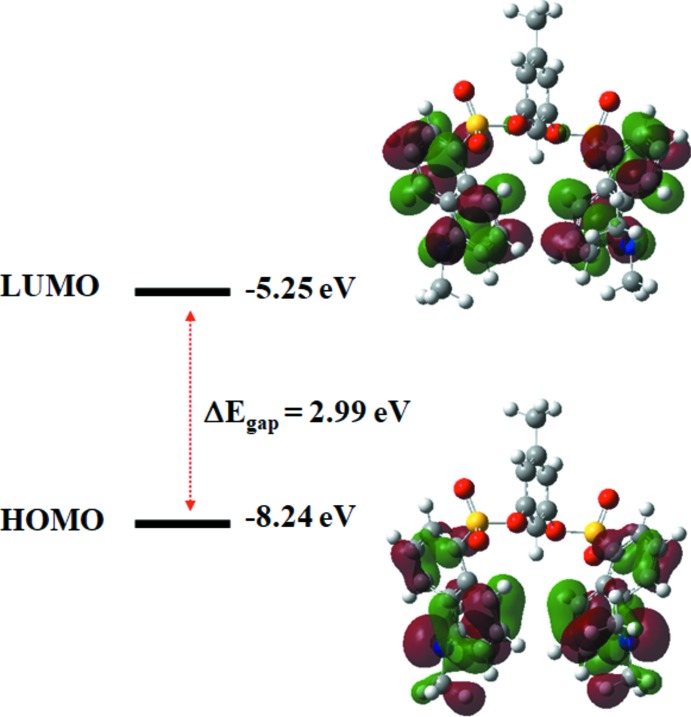
Frontier mol­ecular orbitals of the title compound.

The Density Functional Theory (DFT) calculations were performed at the CAM-B3LYP/6-311G (d,p) level as implemented in the *GAUSSIAN09* program package (Frisch *et al.*, 2009 ▸). The DFT structure optimization of the compound was performed starting from the X-ray geometry. The experimental values of the bond lengths and bond angles match reasonably well with the theoretical values in most cases. However, the lengths of bonds to O atoms involved in hydrogen bonding fit less well, Table 2 ▸. The important features such as conjugation and aromaticity are well illustrated by frontier mol­ecular orbitals. The ionization potential of the mol­ecule is determined from the energy of the highest occupied mol­ecular orbital (HOMO) and the electron affinity is calculated from the energy of the lowest unoccupied mol­ecular orbital (LUMO). The frontier mol­ecular orbital energies, *E*_HOMO_ and *E*_LUMO_ are −8.24 and −5.25 eV, respectively. Insights into the kinetic stability and chemical reactivity of a mol­ecule can be determined from the energy difference between the HOMO and LUMO orbitals, the so-called HOMO–LUMO energy gap. This gap was found here to be 2.99 eV. The HOMO–LUMO energy levels indicate *n*→π* and π→π* transitions and are shown in Fig. 5 ▸. The HOMO is mainly localized on the nitro­gen atom of di­methyl­amine group as well as on the C=C segments of the naphthalene ring systems while the LUMO is located again on the di­methyl­amine substituent and also on the aromatic rings of the naphthalene systems. In Fig. 5 ▸, the negative and positive phases are represented by green and red colours, respectively.

## Database survey   

There are many crystal structures of dansyl derivatives that are similar to the title compound. Two categories of crystal structures of dansyl derivatives are found. The first types are simple organic mol­ecules that pack in the solid state through the many types of inter­molecular inter­actions. For example, in 2-[5-(di­methyl­amino)­naphthalene-1-sulfonamido]­phenyl 5-(di­methyl­amino)­naphthalene-1-sulfonate [CSD (Groom *et al.*, 2016 ▸) refcode NUQDOU; Chainok *et al.*, 2015 ▸), there are two dansyl units connecting to the amine and hydroxyl groups of a 2-amino­phenol, while weak C—H⋯O hydrogen bonds stabilize the crystal structure. In *N*-cyclo­dodecyl-5-(di­methyl­amino)­naphthalene-1-sulfonamide (HODDOU; Fischer *et al.*, 2008 ▸) a cyclo­dodecyl­amine linked to the dansyl substituent adopts a U-shaped conformation, and the crystal packing is stabilized by N—H⋯O hydrogen bonds and C—H⋯π inter­actions between neighbouring mol­ecules. In 8-quinolyl 5-(di­methyl­amino)­naphthalene-1-sulfonate, (DUVFOQ; Xiao & Zhan, 2010 ▸) with an 8-hy­droxy­quinoline ring, C—H⋯O hydrogen bonds and π–π inter­actions between pairs of chains link adjacent mol­ecules. In the crystal structure of *N*-(2-amino­eth­yl)-5-(di­methyl­amino)­naphthalene-1-sulfonamide (BOVBOE; Zhang *et al.*, 2009 ▸) a dansyl compound with a 2-amino­ethyl group, layers are formed through N—H⋯N and weak C—H⋯O hydrogen bonds. In 5,5′-bis­(di­methyl­amino)-*N*,*N*′-(3-methyl-3-aza­pentane-1,5-di­yl)di(naphthalene-1-sulf­onamide) (DABSEH; Horne *et al.*, 2015 ▸), packing in the crystal structure relies on N—H⋯O and C—H⋯O inter­actions.

Metal–dansyl complexes form the second class of common dansyl derivatives. The crystal structures of the di- and trinuclear gold(I) complexes [5-(di­methyl­amino)­naphthalene-1-sulfonamido]­bis­(tri­phenyl­phosphine)digold (UZEJAL) and [5-(di­methyl­amino)­naphthalene-1-sulfonamido]­tris­(tri­phenyl­phosphine)trigold perchlorate (UZEJEP) (Cho *et al.*, 2011 ▸) display weak Au⋯Au inter­actions and C—H⋯π contacts within the mol­ecule. The Pb^2+^ complex 26,28-dibut­oxy-25,27-bis­(*N*-dansylcarbamoylmeth­oxy)-5,11,17,23-tetra­kis(1,1-di­methyl­eth­yl)calix[4]arene (NOJRAG; Buie *et al.*, 2008 ▸), where the calix[4]arene bears two dansylcarboxamide groups, was found to be highly selective and sensitive for the recognition of and coordination to the Pb^2+^ ion.

## Synthesis and crystallization   

The title compound was synthesized by mixing 3,5-di­hydroxy­toluene (1.05 g, 8.46 mmol) and dansyl chloride (4.55g, 17 mmol) using potassium carbonate(2.34g, 17 mmol) as a base in aceto­nitrile solvent (40 ml). The reaction mixture was heated at 363 K and stirred under an N_2_ atmosphere for 24 h. The solvent was removed with a rotary evaporator. The residue was added to water (15 ml) and extracted with di­chloro­methane (3 × 25ml). The organic layer was dried with anhydrous Na_2_SO_4_ and the product was purified by column chromatography using CH_2_Cl_2_ as the eluent. The di­chloro­methane was slowly evaporated to afford a green solid in 65% yield. Light-green block-like crystals were grown in chloro­form at room temperature.

## Refinement   

Crystal data, data collection and structure refinement details are summarized in Table 3 ▸. All H atoms on C were refined using a riding model with *d*(C—H) = 0.95 Å and *U*_iso_(H) = 1.2*U*_eq_(C) for aromatic and *d*(C—H) = 0.98 Å, *U*_iso_(H) = 1.5*U*_eq_(C) for methyl H atoms. As atom Cl lies on a twofold rotation axis, the H atoms of the Cl methyl group are disordered with occupancies fixed at 0.5.

## Supplementary Material

Crystal structure: contains datablock(s) I, global. DOI: 10.1107/S2056989019009058/sj5573sup1.cif

Structure factors: contains datablock(s) I. DOI: 10.1107/S2056989019009058/sj5573Isup2.hkl

Supporting information file. DOI: 10.1107/S2056989019009058/sj5573Isup3.cml

CCDC reference: 1535824

Additional supporting information:  crystallographic information; 3D view; checkCIF report

## Figures and Tables

**Table 1:** Hydrogen-bond geometry (Å, °)

*D*—H⋯*A*	*D*—H	H⋯*A*	*D*⋯*A*	*D*—H⋯*A*
C14—H14⋯O3	0.93	2.49	3.116 (2)	125
C1—H1*B*⋯O3^i^	0.96	2.70	3.528 (2)	145
C9—H9⋯O1^ii^	0.93	2.60	3.486 (2)	158
C16—H16*C*⋯O2^iii^	0.96	2.63	3.475 (2)	147

Symmetry codes: (i) 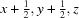; (ii) 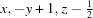; (iii) 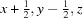.

**Table 2:** Comparison of selected experimental (XRD) bond lengths and angles (Å, °) with those from DFT calculations

Bond/angle	XRD	DFT
S1—O1	1.6006 (12)	1.647
S1—O3	1.4223 (13)	1.453
S1—C6	1.7552 (16)	1.768
O1—C4	1.4166 (17)	1.394
N1—C11	1.413 (2)	1.406
O1—S1—C6	103.11 (7)	103.46
O2—S1—O1	108.81 (7)	108.93
O2—S1—O3	119.32 (9)	119.85
C4—O1—S1	119.05 (9)	119.08
O2—S1—C6	109.18 (8)	109.04

**Table 3:** Experimental details

Crystal data	
Chemical formula	C_31_H_30_N_2_O_6_S_2_
*M*_r_	590.69
Crystal system, space group	Monoclinic, *C*2/*c*
Temperature (K)	296
*a*, *b*, *c* (Å)	15.5072 (6), 12.3504 (5), 16.3017 (5)
β (°)	114.868 (1)
*V* (Å^3^)	2832.62 (18)
*Z*	4
Radiation type	Mo *K*α
μ (mm^−1^)	0.24
Crystal size (mm)	0.44 × 0.44 × 0.4

Data collection	
Diffractometer	Bruker D8 QUEST CMOS
Absorption correction	Multi-scan (*SADABS*; Bruker, 2014 ▸)
*T*_min_, *T*_max_	0.710, 0.745
No. of measured, independent and observed [*I* > 2σ(*I*)] reflections	18169, 2857, 2437
*R*_int_	0.025
(sin θ/λ)_max_ (Å^−1^)	0.625

Refinement	
*R*[*F* ^2^ > 2σ(*F* ^2^)], *wR*(*F* ^2^), *S*	0.037, 0.106, 1.04
No. of reflections	2857
No. of parameters	190
H-atom treatment	H-atom parameters constrained
Δρ_max_, Δρ_min_ (e Å^−3^)	0.23, −0.33

Computer programs: *APEX CCD* and *SAINT* (Bruker, 2013 ▸), *SHELXT* (Sheldrick, 2015*a* ▸), *SHELXL2014* (Sheldrick, 2015*b* ▸), *OLEX2* (Dolomanov *et al.*, 2009 ▸) and *Mercury* (Macrae *et al.*, 2008 ▸).

## supplementary crystallographic information

### Crystal data

**Table:**

C_31_H_30_N_2_O_6_S_2_	*F*(000) = 1240
*M**_r_* = 590.69	*D*_x_ = 1.385 Mg m^−^^3^
Monoclinic, *C*2/*c*	Mo *K*α radiation, λ = 0.71073 Å
*a* = 15.5072 (6) Å	Cell parameters from 8490 reflections
*b* = 12.3504 (5) Å	θ = 3.0–26.4°
*c* = 16.3017 (5) Å	µ = 0.24 mm^−^^1^
β = 114.868 (1)°	*T* = 296 K
*V* = 2832.62 (18) Å^3^	Block, light green
*Z* = 4	0.44 × 0.44 × 0.4 mm

### Data collection

**Table:**

Bruker D8 QUEST CMOS diffractometer	2857 independent reflections
Radiation source: sealed tube	2437 reflections with *I* > 2σ(*I*)
Graphite monochromator	*R*_int_ = 0.025
φ and ω scans	θ_max_ = 26.4°, θ_min_ = 3.0°
Absorption correction: multi-scan (SADABS; Bruker, 2014)	*h* = −19→19
*T*_min_ = 0.710, *T*_max_ = 0.745	*k* = −15→15
18169 measured reflections	*l* = −20→20

### Refinement

**Table:**

Refinement on *F*^2^	Primary atom site location: structure-invariant direct methods
Least-squares matrix: full	Hydrogen site location: inferred from neighbouring sites
*R*[*F*^2^ > 2σ(*F*^2^)] = 0.037	H-atom parameters constrained
*wR*(*F*^2^) = 0.106	*w* = 1/[σ^2^(*F*_o_^2^) + (0.0573*P*)^2^ + 1.5124*P*] where *P* = (*F*_o_^2^ + 2*F*_c_^2^)/3
*S* = 1.04	(Δ/σ)_max_ < 0.001
2857 reflections	Δρ_max_ = 0.23 e Å^−^^3^
190 parameters	Δρ_min_ = −0.33 e Å^−^^3^
0 restraints	

### Special details

**Table:**

Geometry. All esds (except the esd in the dihedral angle between two l.s. planes) are estimated using the full covariance matrix. The cell esds are taken into account individually in the estimation of esds in distances, angles and torsion angles; correlations between esds in cell parameters are only used when they are defined by crystal symmetry. An approximate (isotropic) treatment of cell esds is used for estimating esds involving l.s. planes.

### Fractional atomic coordinates and isotropic or equivalent isotropic displacement parameters (Å^2^)

**Table:**

	*x*	*y*	*z*	*U*_iso_*/*U*_eq_	Occ. (<1)
S1	0.24891 (3)	0.59066 (4)	0.67336 (3)	0.04868 (15)	
O1	0.35036 (8)	0.59425 (9)	0.75828 (7)	0.0458 (3)	
O2	0.21860 (9)	0.69851 (11)	0.64561 (10)	0.0632 (4)	
O3	0.19426 (9)	0.52398 (12)	0.70474 (10)	0.0675 (4)	
N1	0.43181 (10)	0.25203 (12)	0.48853 (10)	0.0535 (4)	
C6	0.27383 (10)	0.52632 (13)	0.58976 (11)	0.0424 (4)	
C7	0.25296 (12)	0.58335 (13)	0.51133 (12)	0.0483 (4)	
H7	0.2260	0.6519	0.5041	0.058*	
C8	0.27244 (12)	0.53779 (15)	0.44241 (12)	0.0526 (4)	
H8	0.2545	0.5739	0.3876	0.063*	
C9	0.31748 (12)	0.44083 (14)	0.45540 (11)	0.0473 (4)	
H9	0.3316	0.4125	0.4096	0.057*	
C10	0.34350 (10)	0.38180 (12)	0.53666 (10)	0.0400 (3)	
C11	0.39393 (11)	0.28115 (13)	0.55059 (11)	0.0458 (4)	
C12	0.40220 (13)	0.21854 (14)	0.62272 (14)	0.0586 (5)	
H12	0.4305	0.1507	0.6302	0.070*	
C13	0.36883 (15)	0.25495 (16)	0.68541 (14)	0.0644 (5)	
H13	0.3740	0.2096	0.7328	0.077*	
C14	0.32938 (13)	0.35391 (15)	0.67920 (12)	0.0532 (4)	
H14	0.3114	0.3778	0.7238	0.064*	
C15	0.31559 (10)	0.42115 (12)	0.60409 (10)	0.0405 (3)	
C16	0.51087 (14)	0.32132 (16)	0.49436 (14)	0.0616 (5)	
H16A	0.4915	0.3958	0.4888	0.092*	
H16B	0.5289	0.3032	0.4465	0.092*	
H16C	0.5640	0.3103	0.5516	0.092*	
C17	0.45713 (16)	0.13831 (16)	0.48903 (16)	0.0728 (6)	
H17A	0.5091	0.1212	0.5457	0.109*	
H17B	0.4755	0.1250	0.4406	0.109*	
H17C	0.4034	0.0939	0.4812	0.109*	
C4	0.42508 (10)	0.65401 (13)	0.75150 (10)	0.0400 (3)	
C3	0.42371 (11)	0.76504 (14)	0.75244 (11)	0.0459 (4)	
H3	0.3723	0.8016	0.7547	0.055*	
C2	0.5000	0.82262 (19)	0.7500	0.0490 (5)	
C5	0.5000	0.59523 (18)	0.7500	0.0398 (5)	
H5	0.5000	0.5199	0.7500	0.048*	
C1	0.5000	0.9447 (2)	0.7500	0.0765 (9)	
H1A	0.4591	0.9706	0.6908	0.115*	0.5
H1B	0.5635	0.9706	0.7663	0.115*	0.5
H1C	0.4774	0.9706	0.7929	0.115*	0.5

### Atomic displacement parameters (Å^2^)

**Table:**

	*U*^11^	*U*^22^	*U*^33^	*U*^12^	*U*^13^	*U*^23^
S1	0.0324 (2)	0.0582 (3)	0.0573 (3)	−0.00268 (16)	0.02067 (19)	−0.00729 (18)
O1	0.0378 (6)	0.0558 (7)	0.0467 (6)	−0.0065 (5)	0.0208 (5)	−0.0008 (5)
O2	0.0455 (6)	0.0600 (8)	0.0765 (9)	0.0133 (6)	0.0182 (6)	−0.0078 (6)
O3	0.0482 (7)	0.0864 (10)	0.0816 (9)	−0.0191 (7)	0.0407 (7)	−0.0157 (7)
N1	0.0461 (8)	0.0486 (8)	0.0618 (9)	0.0003 (6)	0.0189 (7)	−0.0103 (7)
C6	0.0325 (7)	0.0458 (9)	0.0473 (9)	−0.0036 (6)	0.0153 (6)	−0.0010 (7)
C7	0.0429 (8)	0.0426 (9)	0.0548 (10)	0.0020 (7)	0.0158 (8)	0.0066 (7)
C8	0.0509 (9)	0.0554 (10)	0.0452 (9)	0.0027 (8)	0.0142 (8)	0.0142 (7)
C9	0.0451 (9)	0.0538 (10)	0.0411 (8)	−0.0015 (7)	0.0163 (7)	0.0013 (7)
C10	0.0336 (7)	0.0395 (8)	0.0423 (8)	−0.0059 (6)	0.0114 (6)	0.0000 (6)
C11	0.0375 (8)	0.0397 (8)	0.0531 (9)	−0.0052 (6)	0.0121 (7)	−0.0032 (7)
C12	0.0564 (10)	0.0414 (9)	0.0743 (13)	0.0032 (8)	0.0241 (10)	0.0119 (8)
C13	0.0662 (12)	0.0583 (11)	0.0700 (12)	−0.0002 (9)	0.0300 (10)	0.0265 (10)
C14	0.0517 (10)	0.0575 (11)	0.0545 (10)	−0.0016 (8)	0.0264 (8)	0.0113 (8)
C15	0.0336 (7)	0.0425 (8)	0.0433 (8)	−0.0062 (6)	0.0141 (6)	0.0029 (6)
C16	0.0507 (10)	0.0686 (12)	0.0671 (12)	0.0000 (9)	0.0263 (9)	−0.0005 (9)
C17	0.0682 (13)	0.0544 (11)	0.0892 (15)	0.0032 (10)	0.0266 (12)	−0.0176 (10)
C4	0.0332 (7)	0.0499 (9)	0.0360 (8)	−0.0049 (6)	0.0136 (6)	−0.0008 (6)
C3	0.0373 (8)	0.0487 (9)	0.0518 (9)	0.0027 (6)	0.0186 (7)	−0.0020 (7)
C2	0.0446 (12)	0.0440 (12)	0.0577 (14)	0.000	0.0208 (11)	0.000
C5	0.0372 (10)	0.0432 (12)	0.0363 (11)	0.000	0.0129 (9)	0.000
C1	0.0671 (18)	0.0455 (15)	0.124 (3)	0.000	0.0475 (19)	0.000

### Geometric parameters (Å, º)

**Table:**

S1—O1	1.6006 (12)	C13—H13	0.9300
S1—O2	1.4215 (14)	C13—C14	1.352 (3)
S1—O3	1.4223 (13)	C14—H14	0.9300
S1—C6	1.7552 (16)	C14—C15	1.419 (2)
O1—C4	1.4166 (17)	C16—H16A	0.9600
N1—C11	1.413 (2)	C16—H16B	0.9600
N1—C16	1.465 (2)	C16—H16C	0.9600
N1—C17	1.458 (2)	C17—H17A	0.9600
C6—C7	1.374 (2)	C17—H17B	0.9600
C6—C15	1.426 (2)	C17—H17C	0.9600
C7—H7	0.9300	C4—C3	1.372 (2)
C7—C8	1.399 (3)	C4—C5	1.3789 (19)
C8—H8	0.9300	C3—H3	0.9300
C8—C9	1.357 (2)	C3—C2	1.395 (2)
C9—H9	0.9300	C2—C3^i^	1.395 (2)
C9—C10	1.414 (2)	C2—C1	1.507 (3)
C10—C11	1.435 (2)	C5—C4^i^	1.3789 (19)
C10—C15	1.426 (2)	C5—H5	0.9300
C11—C12	1.367 (3)	C1—H1A	0.9600
C12—H12	0.9300	C1—H1B	0.9600
C12—C13	1.400 (3)	C1—H1C	0.9600
			
O1—S1—C6	103.11 (7)	C13—C14—C15	119.53 (17)
O2—S1—O1	108.81 (7)	C15—C14—H14	120.2
O2—S1—O3	119.32 (9)	C10—C15—C6	116.65 (14)
O2—S1—C6	109.18 (8)	C14—C15—C6	124.57 (15)
O3—S1—O1	102.86 (8)	C14—C15—C10	118.77 (15)
O3—S1—C6	112.10 (8)	N1—C16—H16A	109.5
C4—O1—S1	119.05 (9)	N1—C16—H16B	109.5
C11—N1—C16	113.17 (14)	N1—C16—H16C	109.5
C11—N1—C17	115.68 (16)	H16A—C16—H16B	109.5
C17—N1—C16	110.26 (16)	H16A—C16—H16C	109.5
C7—C6—S1	116.60 (13)	H16B—C16—H16C	109.5
C7—C6—C15	122.16 (15)	N1—C17—H17A	109.5
C15—C6—S1	121.22 (12)	N1—C17—H17B	109.5
C6—C7—H7	120.1	N1—C17—H17C	109.5
C6—C7—C8	119.70 (15)	H17A—C17—H17B	109.5
C8—C7—H7	120.1	H17A—C17—H17C	109.5
C7—C8—H8	120.0	H17B—C17—H17C	109.5
C9—C8—C7	120.03 (15)	C3—C4—O1	120.20 (13)
C9—C8—H8	120.0	C3—C4—C5	122.93 (15)
C8—C9—H9	119.1	C5—C4—O1	116.74 (14)
C8—C9—C10	121.77 (16)	C4—C3—H3	120.3
C10—C9—H9	119.1	C4—C3—C2	119.48 (16)
C9—C10—C11	121.12 (15)	C2—C3—H3	120.3
C9—C10—C15	119.14 (14)	C3—C2—C3^i^	118.7 (2)
C15—C10—C11	119.69 (14)	C3^i^—C2—C1	120.64 (11)
N1—C11—C10	118.03 (15)	C3—C2—C1	120.65 (11)
C12—C11—N1	123.69 (16)	C4—C5—C4^i^	116.5 (2)
C12—C11—C10	118.28 (16)	C4—C5—H5	121.8
C11—C12—H12	119.5	C4^i^—C5—H5	121.8
C11—C12—C13	121.09 (17)	C2—C1—H1A	109.5
C13—C12—H12	119.5	C2—C1—H1B	109.5
C12—C13—H13	119.0	C2—C1—H1C	109.5
C14—C13—C12	122.05 (17)	H1A—C1—H1B	109.5
C14—C13—H13	119.0	H1A—C1—H1C	109.5
C13—C14—H14	120.2	H1B—C1—H1C	109.5
			
S1—O1—C4—C3	72.98 (16)	C9—C10—C11—N1	11.1 (2)
S1—O1—C4—C5	−111.07 (11)	C9—C10—C11—C12	−168.50 (16)
S1—C6—C7—C8	178.93 (13)	C9—C10—C15—C6	−8.3 (2)
S1—C6—C15—C10	−172.59 (11)	C9—C10—C15—C14	170.72 (15)
S1—C6—C15—C14	8.4 (2)	C10—C11—C12—C13	−4.8 (3)
O1—S1—C6—C7	−121.15 (13)	C11—C10—C15—C6	174.23 (13)
O1—S1—C6—C15	57.51 (13)	C11—C10—C15—C14	−6.7 (2)
O1—C4—C3—C2	176.99 (11)	C11—C12—C13—C14	−1.7 (3)
O1—C4—C5—C4^i^	−176.50 (14)	C12—C13—C14—C15	4.0 (3)
O2—S1—O1—C4	−53.02 (13)	C13—C14—C15—C6	179.28 (16)
O2—S1—C6—C7	−5.58 (15)	C13—C14—C15—C10	0.3 (2)
O2—S1—C6—C15	173.07 (12)	C15—C6—C7—C8	0.3 (2)
O3—S1—O1—C4	179.51 (11)	C15—C10—C11—N1	−171.45 (13)
O3—S1—C6—C7	128.91 (14)	C15—C10—C11—C12	8.9 (2)
O3—S1—C6—C15	−52.44 (15)	C16—N1—C11—C10	68.68 (19)
N1—C11—C12—C13	175.62 (17)	C16—N1—C11—C12	−111.71 (19)
C6—S1—O1—C4	62.80 (12)	C17—N1—C11—C10	−162.74 (15)
C6—C7—C8—C9	−4.3 (3)	C17—N1—C11—C12	16.9 (2)
C7—C6—C15—C10	6.0 (2)	C4—C3—C2—C3^i^	−0.62 (10)
C7—C6—C15—C14	−172.98 (16)	C4—C3—C2—C1	179.38 (10)
C7—C8—C9—C10	1.8 (3)	C3—C4—C5—C4^i^	−0.66 (11)
C8—C9—C10—C11	−177.88 (15)	C5—C4—C3—C2	1.3 (2)
C8—C9—C10—C15	4.7 (2)		

Symmetry code: (i) −*x*+1, *y*, −*z*+3/2.

### Hydrogen-bond geometry (Å, º)

**Table:**

*D*—H···*A*	*D*—H	H···*A*	*D*···*A*	*D*—H···*A*
C14—H14···O3	0.93	2.49	3.116 (2)	125
C1—H1*B*···O3^ii^	0.96	2.70	3.528 (2)	145
C9—H9···O1^iii^	0.93	2.60	3.486 (2)	158
C16—H16*C*···O2^iv^	0.96	2.63	3.475 (2)	147

Symmetry codes: (ii) *x*+1/2, *y*+1/2, *z*; (iii) *x*, −*y*+1, *z*−1/2; (iv) *x*+1/2, *y*−1/2, *z*.
